# Disseminated Blastomycosis in an Immunocompetent Patient

**DOI:** 10.7759/cureus.30391

**Published:** 2022-10-17

**Authors:** Ahmed H Abdelfattah, Sania Ayub, Maryam Talib, Hadeel Dawoud, Usama Talib

**Affiliations:** 1 Internal Medicine, University of Kentucky College of Medicine, Lexington, USA; 2 Internal Medicine, Quetta Institute of Medical Sciences, Quetta, PAK; 3 Internal Medicine, Basic Health Unit, Hajiwala, PAK; 4 Critical Care Medicine, Mansoura University, Mansoura, EGY

**Keywords:** cutaneous fungal infections, fungal infection, skin blastomycosis, pulmonary blastomycosis, blastomycosis diagnosis, disseminated blastomycosis

## Abstract

Blastomycosis is caused by Blastomyces dermatitidis, which is endemic in certain areas in North America. It usually causes lung infection, and it can disseminate to other organs in immunocompromised individuals. Common sites for dissemination include skin, central nervous system (CNS), and bone. Dermatological spread is the commonest site for extrapulmonary spread. The diagnosis can be easily missed due to nonspecific presentation and variable dermatological presentations. Treatment is necessary even if the patient has improvement in symptoms without previous treatment. We present a case of disseminated blastomycosis in a 40-year-old male without known risk factors that went undiagnosed for over a year.

## Introduction

Blastomycosis is a fungal infection caused by Blastomyces dermatitidis, a dimorphic fungus endemic in the Ohio and Mississippi River valleys, the Great Lakes region, and the southeastern United States [1]. Pulmonary infection is the most common presentation following inhalation of the spores, however, Blastomycosis can range from asymptomatic infection to fulminant infection with acute respiratory distress syndrome (ARDS) and death [1,2]. Extrapulmonary disease occurs following hematogenous spread, with the skin being the most common site of extrapulmonary disease [1,2]. In descending order, skin, bone, prostate, and central nervous system (CNS) involvements are the most reported manifestations of extrapulmonary blastomycosis [3]. Respiratory failure is the most common cause of death in blastomycosis, hence the treatment should be considered in all patients with blastomycosis to prevent extrapulmonary dissemination [4]. We present a case of disseminated blastomycosis in a 40-year-old male.  

## Case presentation

A 40-year-old male with a history of opiate use disorder and chronic untreated hepatitis C without evidence of liver cirrhosis presented to the hospital with persistent wounds on his head, hands, right shin, and left foot for almost one-year duration. He saw a local dermatologist who took a biopsy for skin culture four weeks prior to presentation. The patient reported that one year back he was seen in the ED for increased shortness of air and coughing up blood, at the time he was diagnosed and treated for community-acquired pneumonia (CAP). At the time of his initial symptoms, he had been working in the crawl space under his home for a few months to address plumbing-related issues. He was also frequently hunting outdoors in Kentucky. He denied foreign travel or travel out of state, incarceration, zoonotic exposure, or illicit or intravenous drug use (IVDU). He continued to cough up a small amount of bright red blood (BRB) daily for several months after his admission for CAP. 

**Figure 1:**
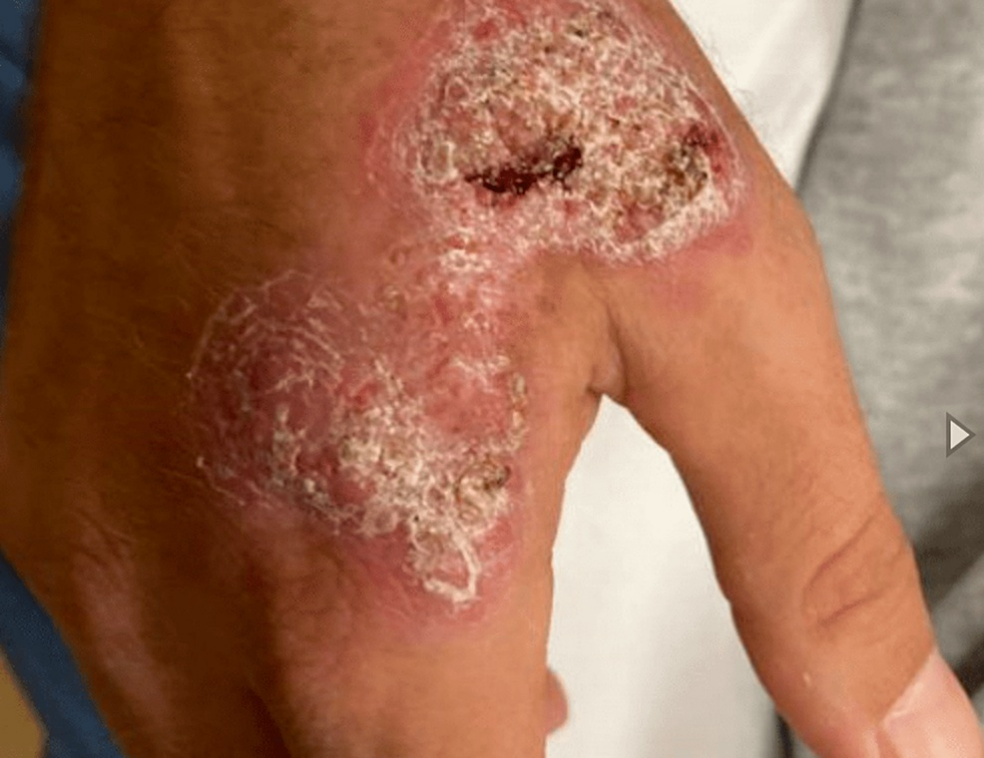
Right hand wart-like lesions.

Three to four months after initial hospitalization, he developed "cysts" on his right hand (Figure 1) and anterior right shin which eventually burst open, draining purulent foul-smelling discharge.

**Figure 2:**
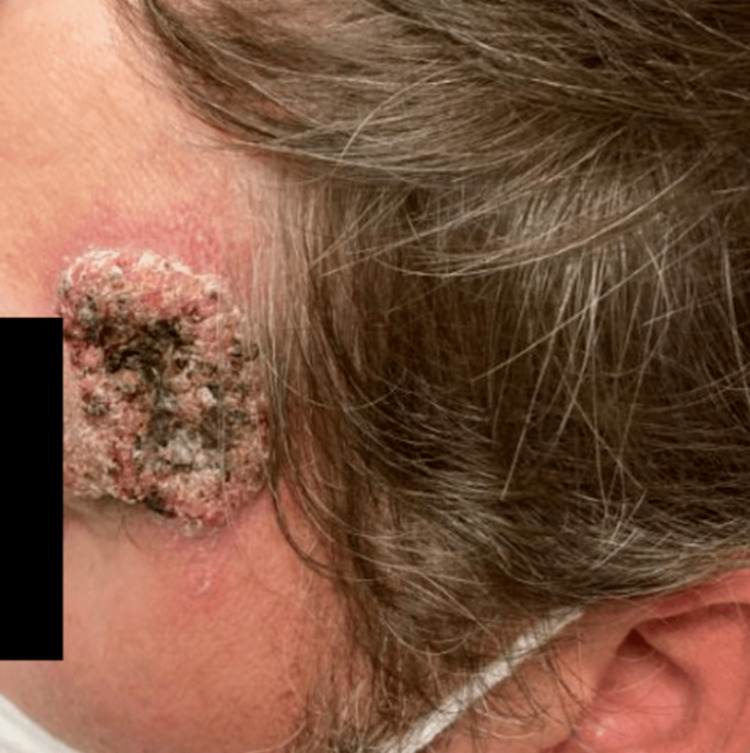
Forehead wart-like lesion.

**Figure 3:**
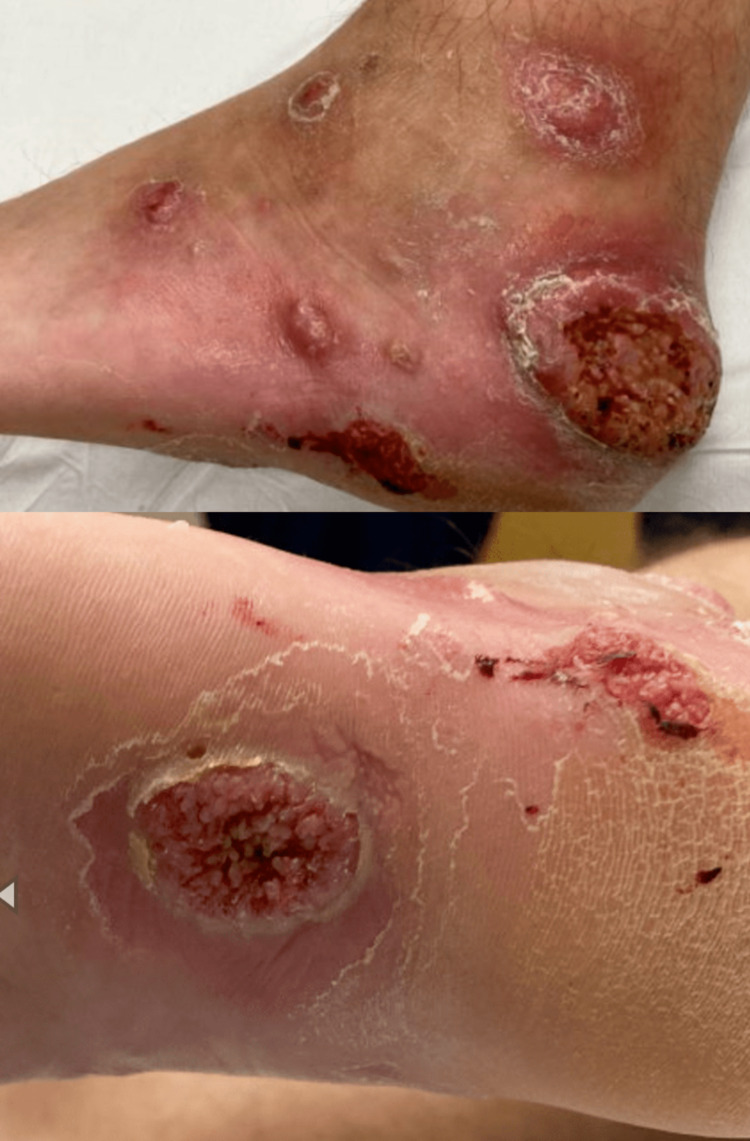
Left foot lesions.

The wounds then developed a “scab with wart-like appearance.” The patient then developed similar ulcerated lesions on the left side of his forehead (Figure 2) and left foot (Figure 3). He saw his PCP and was seen in the ED and completed several courses of oral antibiotics without improvement.

Four weeks prior to admission he was seen by a dermatology clinic where a punch biopsy was collected on all wounds. He did not receive any results for four weeks and given the skin lesions were not resolving he presented to our hospital. He was admitted for infectious disease (ID) and dermatology evaluation. Vital signs were stable on ED arrival. Laboratory tests were mostly unremarkable (Table 1).

**Table 1:** Laboratory investigations. CRP: C-reactive protein, ESR: erythrocyte sedimentation rate, PCR: polymerase chain reaction, RSV: respiratory syncytial virus, RPR: rapid plasma reagin, EIA: enzyme immunoassay, AFB: acid-fast bacilli

Laboratory Investigations	Value	Reference Range
White blood cells	Normal	3.70 - 10.30 10*3/uL
CRP	Increased	<8.0 mg/dL
ESR	Increased	<15.0 mm/hr
PCR for COVID-19, Influenza A&B, RSV	Negative	Negative
Blastomycosis antibody	Increased	<=0.9 IV
QuantiFERON gold	Non - Reactive	Non - Reactive
Fungal and isolator blood cultures	Negative	Negative
Chlamydia	Non - Reactive	Non - Reactive
RPR	Non - Reactive	Non - Reactive
Histoplasma urine and serum antigen	Not detected	Not detected
Neisseria	Not detected	Not detected
Coccidioides Quantitative Antigen EIA	Not detected	Not detected
HIV	Non - Reactive	Non - Reactive
Hepatitis C viral load	Increased	< 12 IU/mL
Sputum AFB	Negative	Negative

On examination, he had rhonchi in the left upper lung. He also had dry, erythematous, and spongy lesions on the left foot (calcaneus and ventral foot), left forehead, right anterior shin, and right hand. Chest X-ray showed no acute findings. X-ray of the Left foot showed no bony abnormality or appreciable soft tissue gas. CT head showed a 3 cm left anterior temporal scalp lesion with mild to moderate sinus opacification. CT chest showed left upper lung para-mediastinal small patch of dense consolidation versus atelectasis. MRI of the head showed no concern of intracranial involvement or extension of the lesion and no acute infarct/mass.

The outpatient dermatology had only sent a skin culture which had not grown anything per communication. Biopsy from forehead skin lesion per recommendation from dermatopathologist was sent for culture and pathology. The culture showed broad budding yeast and was later confirmed as blastomycosis. Pathology was consistent with granulomatous dermatitis with reactive squamous hyperplasia and Grocott methenamine silver (GMS) stain positive for yeast forms compatible with Blastomyces species. Blastomycosis antibody was elevated. Laboratory investigations during hospitalization have been listed in Table 1. He was diagnosed with disseminated blastomycosis. ID evaluated the patient, and lumbar puncture (LP) was discussed but it was unnecessary based on the mental status and MRI findings. He was started on amphotericin B for 10 days and discharged with liquid daily itraconazole for six to 12 months with a plan for dermatology, ID, and PCP follow-up for itraconazole levels and liver enzymes. His skin lesions had started to heal by the time he was discharged from the hospital.

## Discussion

Blastomycosis is a systemic fungal infection. B. dermatitidis is the most common organism in North America responsible for blastomycosis. It usually spreads by inhalation and produces primary lung lesions [1,2]. It can spread through a hematogenous route to other organs such as skin, CNS, and bone [3]. Pulmonary involvement in blastomycosis can range from asymptomatic infection to fulminant fatal infection [1,2]. It is reported that dissemination of the infection can occur in up to 30% of symptomatic patients [2]. Dust clouds and soil exposure in an endemic area are risk factors for acquiring the infection [5]. Our patient reported plumbing-related activities in the basement of his house and had outdoor hunting trips prior to developing symptoms. These activities might have led to the possible exposure.

Infection with blastomycosis and its dissemination is common in immunosuppressed conditions and being an outdoor worker [6]. Our patient reported outdoor activity as mentioned above in the presentation and he did not have an immunosuppressed state.

Diagnosis of blastomycosis is difficult due to nonspecific initial presentation. Most cases are asymptomatic, the rest may show mild to severe respiratory findings that can resemble, pneumonia, tuberculosis, or another systemic mycosis in endemic regions with associated skin lesions including verrucous and ulcerative forms [7]. Skin lesions can be found in almost 40% to 80% of the symptomatic cases [7]. Laboratory diagnosis can be made by direct visualization of the yeast and a positive culture, also well-matched histology is often needed for presumptive diagnosis and treatment initiation [8].

Treatment of blastomycosis is necessary for all patients irrespective of the resolution of symptoms before initiating the treatment [9]. Treatment also depends on the site involved and the extent of the infection [9]. In moderate to severe cases treatment is initiated with amphotericin B followed by prolonged course with itraconazole [10]. Case fatality from blastomycosis is up to 78% without treatment with antifungal medications, hence reinforcing the need for appropriate treatment [7].

## Conclusions

This case highlights the clinical manifestations of disseminated blastomycosis and the need to be kept in differentials in patients without known predisposing conditions. We also stress the importance of maintaining a high index of suspicion in individuals presenting with unexplainable, prolonged, vague symptoms, who are residents of an endemic region. Diagnosis is important to initiate appropriate treatment and prevent CNS or other organ involvement and minimize case fatality related to blastomycosis. 
